# Reprogramming induced by isoliquiritigenin diminishes melanoma cachexia through mTORC2-AKT-GSK3β signaling

**DOI:** 10.18632/oncotarget.16655

**Published:** 2017-03-29

**Authors:** Xiao-Yu Chen, De-Fang Li, Ji-Chun Han, Bo Wang, Zheng-Ping Dong, Li-Na Yu, Zhao-Hai Pan, Chuan-Jun Qu, Ying Chen, Shi-Guo Sun, Qiu-Sheng Zheng

**Affiliations:** ^1^ College of Chemistry & Pharmacy, Northwest A&F University, Yangling, Shaanxi, 712100, China; ^2^ Binzhou Medical University, Yantai, Shandong, 264003, China; ^3^ Key Laboratory of Xinjiang Endemic Phytomedicine Resources of Ministry of Education, School of Pharmacy, Shihezi University, Shihezi, 832002, China

**Keywords:** reprogramming, melanoma, cachexia, isoliquiritigenin, mTORC2-AKT-GSK3β signaling

## Abstract

Isoliquiritigenin (ISL), a member of the flavonoids, is known to have anti-tumor activity *in vitro* and *in vivo*. The effect of ISL on reprogramming in cancer cells, however, remains elusive. In this study, we investigated the effect of ISL on reprogramming in human melanoma A375 cells. ISL (15 μg/ml) significantly inhibited A375 cell proliferation, anchorage independent cell proliferation and G2/M cell cycle arrest after ISL exposure for 24 h. However, there were no significant changes in apoptosis rate. Terminal differentiation indicators (melanin content, melanogenesis mRNA expression, tyrosinase (TYR) activity) were all up-regulated by ISL treatment. In ISL-treated cells, glucose uptake, lactate levels and mRNA expression levels of GLUT1 and HK2 were significantly decreased, and accompanied by an increase in O_2_ consumption rate (OCR) and adenosine triphosphate (ATP) deficiency. Protein expression levels of mTORC2-AKT-GSK3β signaling pathway components (mTOR, p-mTOR, RICTOR, p-AKT, p-GSK3β) decreased significantly after ISL treatment. Co-treatment of ISL and the mTOR-specific inhibitor Ku-0063794 had a synergistic effect on the inhibition of proliferation, and increased melanin content and TYR activity. Glucose uptake and lactate levels decreased more significantly than treatment with ISL alone. These findings indicate that ISL induced reprogramming in A375 melanoma cells by activating mTORC2-AKT-GSK3β signaling.

## INTRODUCTION

The nucleus of somatic cells can be ‘reprogrammed’ to exhibit embryonic stem cell (ESC)-like pluripotent differentiation properties by various means [1]. Cancer cells have been reprogrammed in a similar fashion to various differentiation lineages, such as undifferentiated ESCs, or terminally differentiated ESCs with concomitant abrogation of tumorigenicity [2, 3]. Reprogramming cancer cells has been shown to reduce the proliferative potential of human colon cancer cells [4], non-small cell lung cancer cells [5] and breast cancer cells [6].

Metabolic processes can also be reprogrammed. Normal cells rely on a process called oxidative phosphorylation (OXPHOS) [7]. However, cancer cell metabolism has been described to undergo “metabolic reprogramming”. This reprogramming results in a higher rate of glycolysis and an increase in lactate secretion despite the presence of oxygen, a phenomenon known as the Warburg effect, which is described as a “hallmark of cancer” [7, 8]. Current studies describe metabolic reprogramming as a central player in malignancy and proliferation. Decreased metabolic reprogramming diminishes survival in multiple pancreatic cancer cell lines [9], and inhibits the proliferation of Panc-1 human pancreatic cancer cells [10].

mTOR is the catalytic subunit of two molecular complexes: mTOR Complex 1 (mTORC1) and Complex 2 (mTORC2) that have distinct substrate specificities, are differentially sensitive to rapamycin, and are differentially regulated [11, 12]. mTOR Complex 1 (mTORC1/RAPTOR) responds to growth signals and nutrients and mTOR Complex 2 (mTORC2/RICTOR) primarily responds to growth signals [13, 14]. Recent studies suggest that mTORC2 has a central function in metabolic reprogramming, thereby contributing to glioblastoma growth and drug resistance [15]. mTORC2 appears to control the metabolic reprogramming of cancer cells in at least three ways: by modulating import of nutrients (glucose, lipids, amino acids), through regulation of the activity or expression of specific metabolic enzymes, and by the rewiring metabolic networks [15]. Recent reports have shown that mTORC2 promoted T cell [16], osteoblast [17] and C2C12 myoblast [18] differentiation, and inhibited cancer cell growth [19–21].

Isoliquritigenin (ISL) is an abundant dietary flavonoid with a chalcone structure, which is an important constituent in Glycyrrhizae Radix (GR). ISL has been shown to have anti-tumor activity *in vitro* and *in vivo* [22–24]. Previously, we reported that ISL induced cancer cell differentiation [25, 26], and reduced glycolysis in a mouse melanoma cell line [27, 28]. Despite these advances, very little is known about the pharmacological mechanism of action of ISL in human melanoma cell lines. We hypothesized that ISL might reprogram human melanoma cell lines into terminal differentiation phenotypes by altering metabolic activity through mTOR2 signaling. In addition, we explored ISL's potential therapeutic mechanisms in A375 human melanoma cell.

## RESULTS

### ISL inhibited A375 cell proliferation and induced A375 cell cycle arrest in G2/M

**Figure 1:**
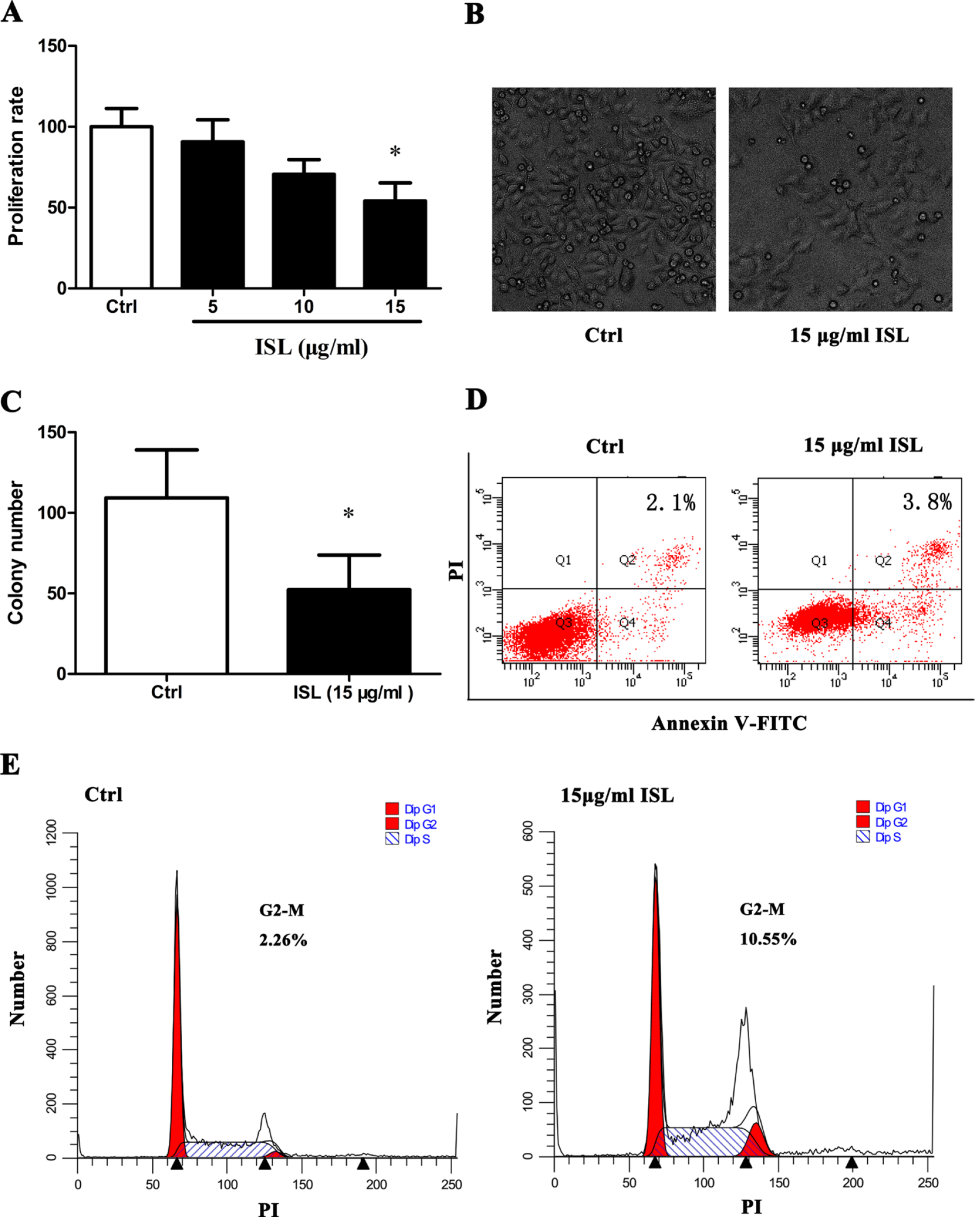
ISL inhibited A375 melanoma cell proliferation (**A**) Cell proliferation rate evaluated by MTT assay. (**B**) Phase-contrast micrographs (200×) showed morphological changes in ISL-treated A375 cells. (**C**) Anchorage independent cell growth measured by colony formation assay. Results represent the average number of colonies/field (see supplementary material). (**D**) Apoptosis in A375 cells treated with ISL, as measured by flow cytometric analysis of cells co-stained with Annexin V and PI. (**E**) Accumulation of cells in G2/M phase following ISL treatment determined by flow cytometry. (A, C) Bars represent mean ± SD of three independent experiments. *P < 0.05 versus control.

After 24 h of exposure, ISL treatment decreased proliferation to 56% compared to control cells (*P* < 0.05) in a concentration- and time-dependent manner (Figure 1A). In addition, ISL treatment induced morphological changes that are shown in phase-contrast micrographs (Figure 1B). Figure 1B shows a decreased cell number in ISL-treated cells compared with controls, and ISL-treated cells were notably larger in size than control cells. The decrease in cell number was accompanied by a 2-fold decrease in the number of colonies, as measured by the colony formation assay (Figure 1C, Supplementary Figure 1). However, no significant differences were observed in the apoptosis rate between ISL-treated and control cells, with early apoptosis rates of 2.1% and 3.8% in control or ISL-treated cells, respectively (Figure 1D). The percentage of ISL-treated cells in the G2/M phase as measured by flow cytometry was 10.55% compared to 2.26% in control cells with a statistically significant (*P* < 0.05) (Figure 1E).

### ISL induced cell differentiation in human melanoma A375 cells

**Figure 2:**
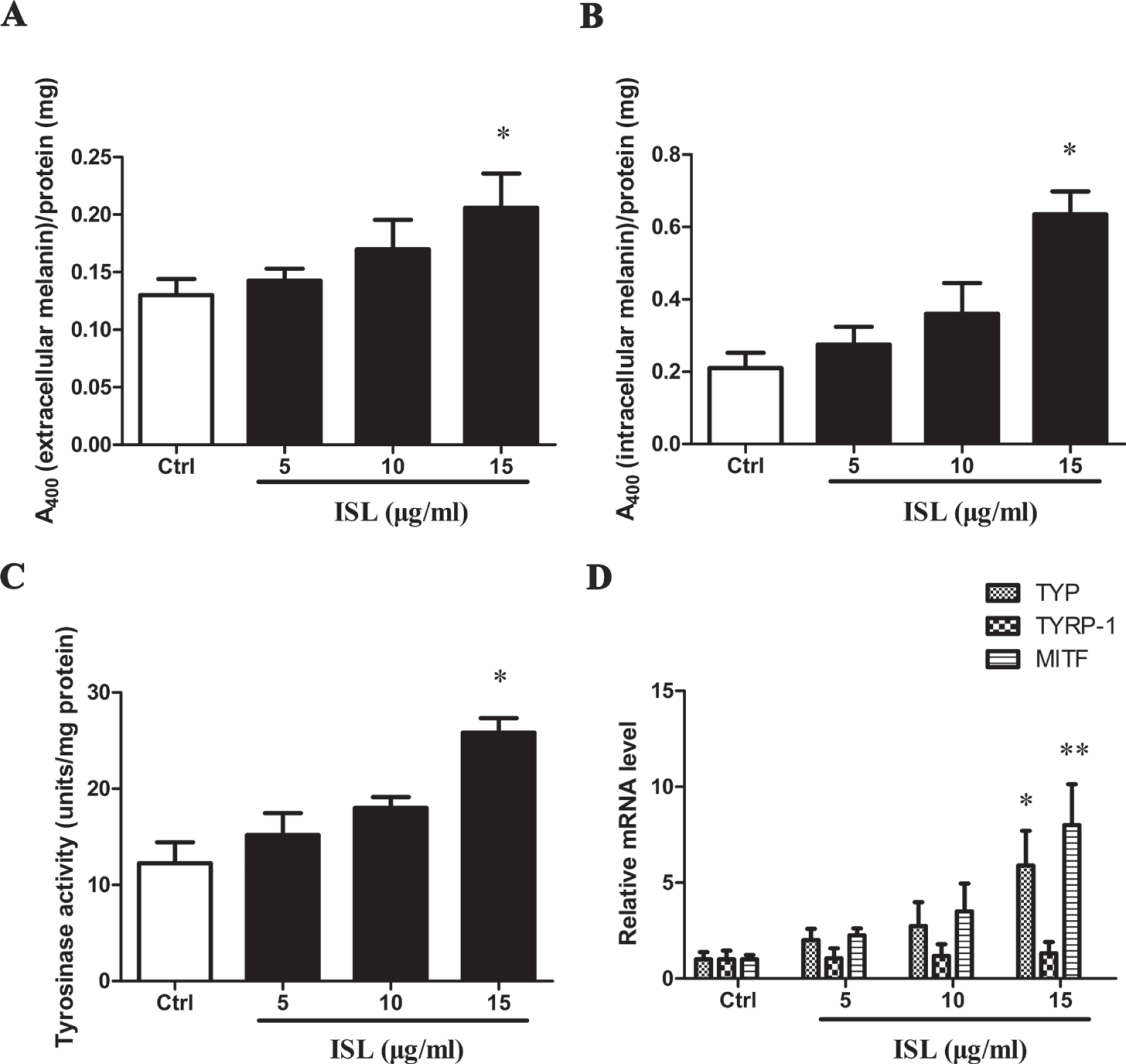
ISL induced melanoma cell differentiation Melanin content in ISL-treated A375 cells measured at 400 nM. (**A**) Extracellular melanin. (**B**) Intracellular melanin. (**C**) Effect of ISL treatment on tyrosinase activity in A375 cells. (**D**) Real-time qPCR analysis for melanogenesis gene expression in A375 cells treated with ISL. Bars represent the mean ± SD of three independent experiments; *P < 0.05, **P < 0.01 versus control.

Our study shows a dose-dependent increase in extracellular (Figure 2A) and intracellular (Figure 2B) melanin content following treatment with ISL, with statistically significant increases using 15 μg/ml of ISL. TYR activity increased significantly after treatment with ISL for 24 h (Figure 2C). In addition, TYR mRNA expression (*P* < 0.05) and MITF (microphthalmia-associated transcription factor) (*P* < 0.01) significantly increased in the ISL-treated group (Figure 2D).

### ISL decreased glycolysis and induced ATP depletion in A375 cells

**Figure 3:**
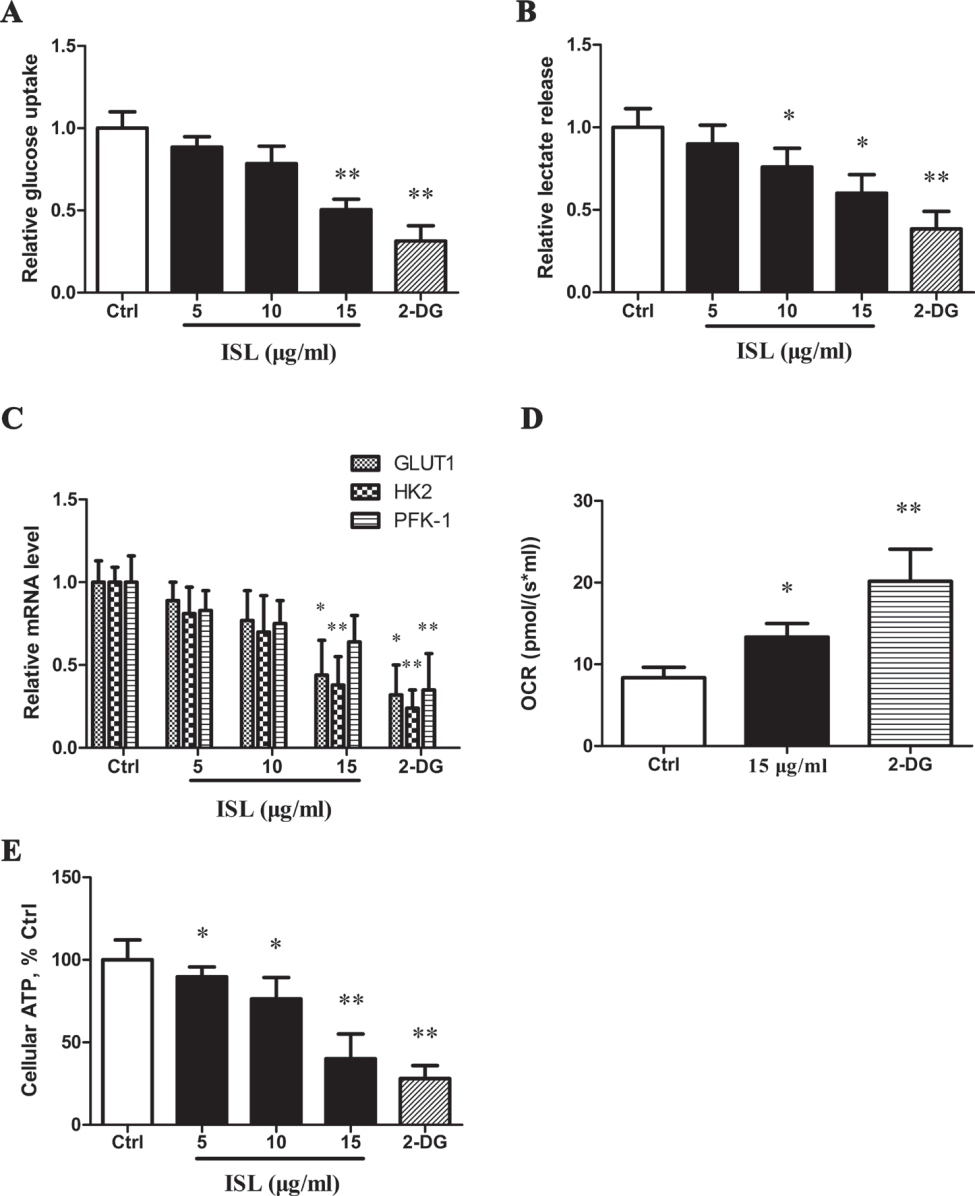
ISL decreased glycolysis and induces ATP depletion (**A**) Analysis of glucose uptake. (**B**) Analysis of lactic release. (**C**) Real-time qPCR analysis for glycolysis gene expression. (**D**) Cellular O_2_ was calculated as the time derivative of the oxygen content in the chamber, and results represent pmol/(s*ml). (**E**) Determination of cellular ATP level in ISL-treated A375 cells. Bars represent the mean ± SD of three independent experiments; *P < 0.05, **P < 0.01 versus control. 2-DG served as a glycolysis inhibitor.

Treatment of A375 cells with ISL resulted in a decrease of glucose uptake (Figure 3A) and release of lactate (Figure 3B) in a concentration-dependent manner. As a positive control, we used 2-deoxy-D-glucose (2-DG), a known inhibitor of glycolysis, via competitive inhibition after phosphorylation by hexokinase [29], and found a significant greater inhibition of glucose uptake and lactate release. To determine the mechanism of action, we evaluated the expression of genes encoding glucose transporter-1 (GLUT1) and the glycolytic enzymes hexokinase II (HK2) and phosphofructokinase (PFK-1). GLUT1 and HK2 expression were reduced in cells treated with ISL (Figure 3C, Supplementary Table 1), while 2-DG caused a marked decrease the expression of all three key glycolysis genes.

In A375 cells treated with 15 μg/ml ISL or 2-DG, the cellular oxygen consumption rate (OCR) was determined. ISL induced a significant increase in OCR (14.345 pmol/(s* ml), *P* < 0.05) compared to control cells (8.365 pmol/(s* ml)), and 2-DG increased the OCR to a greater degree (Figure 3D). All concentrations of ISL significantly depleted ATP levels in a dose-dependent manner, and 2-DG treatment resulted in the lowest cellular ATP level (Figure 3E).

### ISL induced melanoma reprogramming via mTOR2-AKT- GSK3β signaling

**Figure 4:**
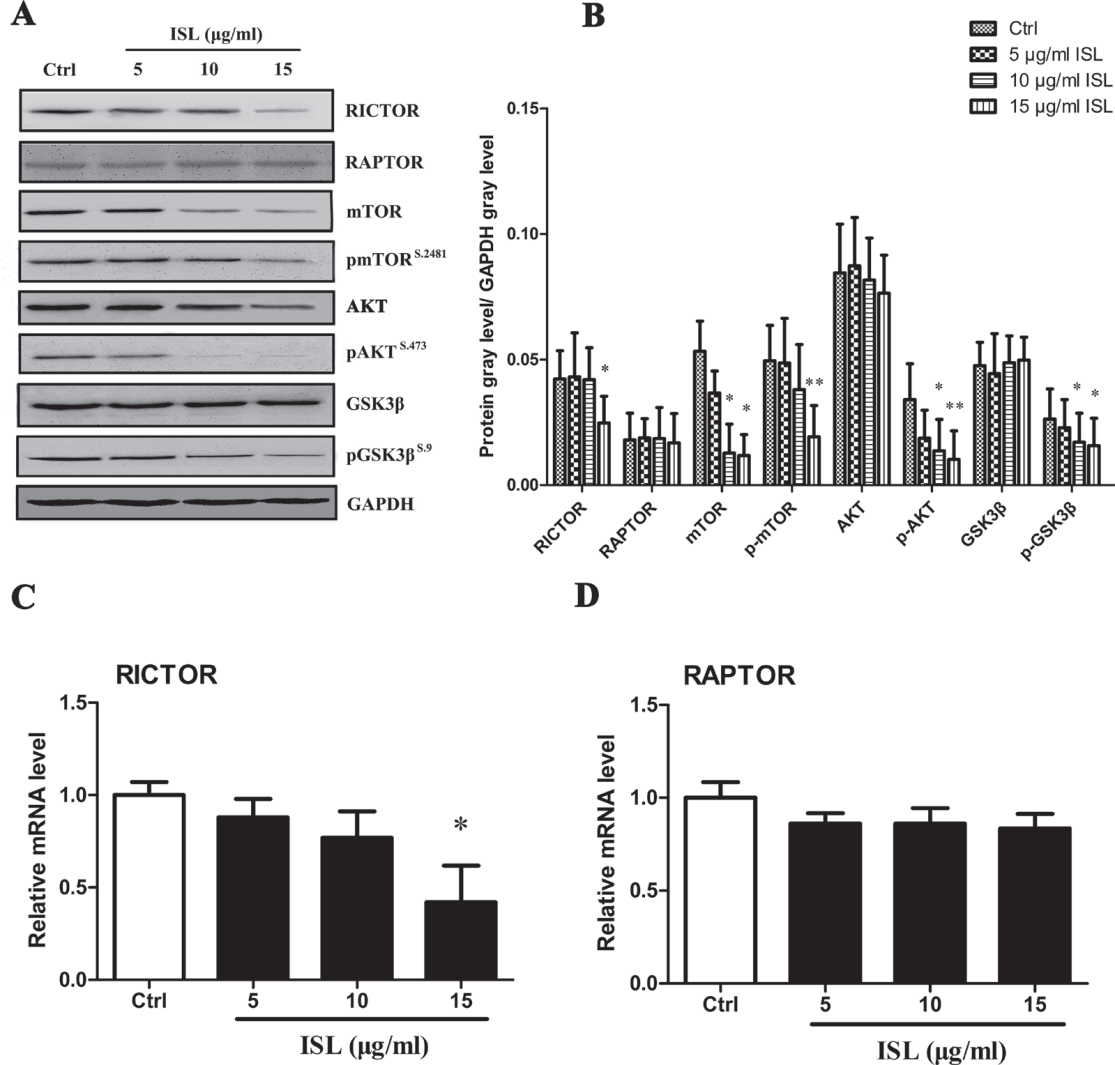
ISL treatment activated mTOR2-AKT-GSK3β signaling (**A**) Western blotting analysis for activated and total AKT or GSK3β, mTOR, and mTOR2 and mTOR1 dependent proteins RICTOR and RAPTOR. (**B**) qPCR analysis of relative mRNA expression of RICTOR and RAPTOR. Bars represent the mean ± SD of three independent experiments; *P < 0.05, **P < 0.01 versus control.

Western blot analysis was used to determine the levels of mTOR, the mTOR2-dependent protein RICTOR and downstream AKT, GSK3β (Figure 4A, 4B). Treatment for 24 hours with 15 μg/ml ISL modestly reduced the expression of mTOR and RICTOR, whereas the expression of RAPTOR was not significantly altered. The level of p-AKT (Ser473) was significantly decreased in 15 μg/ml ISL-treated cells, with no significant differences in total AKT levels. The phosphorylated form of GSK3β was significantly decreased by ISL treatment, with no significant differences in total expression levels of GSK3β.

To further characterize the involvement of ISL on the regulation of the mTOR2 pathway, qPCR was performed to determine mRNA levels. ISL (15 μg/ml) significantly decreased the mRNA expression of RICTOR (Figure 4C, Supplementary Table 1), but had no significant effect on RAPTOR mRNA expression (Figure 4D, Supplementary Table 1).

**Figure 5:**
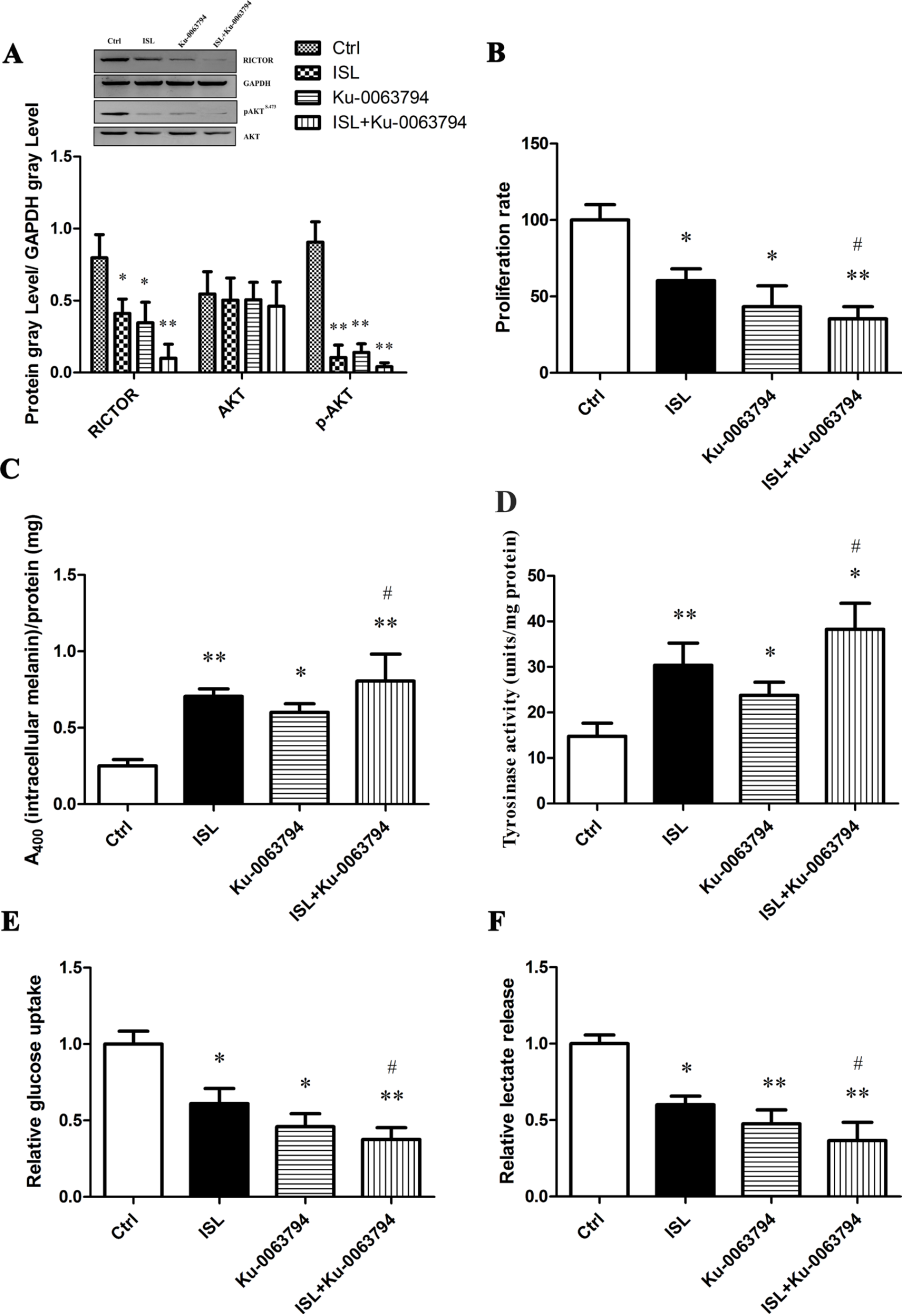
The effect of ISL on mTOR2-AKT-GSK3β signaling using mTOR specific inhibitor Ku-0063794 All assays were performed after A375 cells were treated for 24 hours with 15 μg/ml ISL, the mTOR-specific inhibitor-Ku-0063794 (1 μM), or both drugs simultaneously. (**A**) Western blot analysis for the mTOR2-dependent protein RICTOR, activated AKT and total AKT. (**B**) Cell proliferation rate determined by MTT assay. (**C**) Intracellular melanin content. (**D**) Tyrosinase activity. (**E**) Analysis of glucose uptake. (**F**) Analysis of lactate release in ISL-treated A375 cells in the absence or presence of Ku-0063794. *P < 0.05, **P < 0.01 versus control; ^#^*P* < 0.05 versus ISL treatment.

We used the mTOR-specific inhibitor, Ku-0063794 to further characterize the effects of ISL on protein expression and phosphorylation of members of the mTOR pathway. ISL or Ku-0063794 (1 μM) significantly decreased the protein expression of RICTOR and pAKT, without a significant change in total AKT levels (Figure 5A). When these compounds were given simultaneously, effects were synergistic. ISL and Ku-0063794 co-treatment also led to a significant inhibition of A375 cell proliferation (Figure 5B). Both ISL and Ku-0063794 increased intracellular melanin and tyrosinase activity when given alone, and co-treatment was synergistic (Figure 5C, 5D). In addition, Ku-0063794 treatment resulted in a decrease in glucose uptake (Figure 5E) and release of lactate (Figure 5F); these effects were synergistic when given in conjunction with ISL (*P* < 0.05) (Figure 5E, 5F).

## DISCUSSION

Cancer cells have the potential to be “reprogrammed” and undergo terminal differentiation into various cell types when given the appropriate stimuli. Zhang, et al. reprogrammed sarcoma cells to differentiate into mature connective tissue and red blood cells; these terminally differentiated cells irreversibly lost their tumorigenic potential [3]. It is unclear whether other types of cancer cells can be reprogrammed by different approaches for dedifferentiation or terminal differentiation. This is the basis of acute promyelocytic leukemia therapy with all-trans retinoic acid, which induces terminal differentiation of leukemic promyelocytes into ‘normal cells’ [30].

Identifying methods to induce terminal differentiation in solid tumors is challenging. In our previous studies, we demonstrated that differentiation of mouse melanoma cells induced by ISL was responsible for diminishing cancer cell cachexia [26]. We also found that ISL altered melanin anabolism and glycolysis of mouse melanoma cells in our recent work [27, 28]. In this study, we hypothesized that ISL was capable of inducing human melanoma reprogramming and we investigated the underlying mechanism of action.

We analyzed the effects of ISL on cell proliferation in human melanoma cells, and found a significant decrease in the overall proliferation rate and the anchorage-dependent rate. However, the decrease in proliferation was not accompanied by increased apoptosis rates, and we found that the cell cycle was blocked in the G2/M phase. We therefore conclude that the inhibition of proliferation was not caused by apoptosis.

We hypothesize that the decrease in proliferation in the absence of increased apoptosis was due to terminal differentiation, and measured melanin content and melanogenesis parameters (tyrosinase activity and TYRP1 expression) to determine if terminal differentiation had occurred in A375 cells. In clinical studies, tyrosinase activity and TYRP1 expression have been shown to correlate inversely with the tumor stage [31]. The activation of the melanogenic pathway (melanin content, tyrosinase activity and TYRP1 expression) was determined after ISL treatment. MITF is a master gene regulating differentiation of melanocytes, and a lineage survival oncogene mediating pro-proliferative function in malignant melanoma [32]. We found an increased expression of MITF after ISL stimulation. Taken together, these results suggest that ISL treatment induces terminal differentiation in melanoma cells.

For most of their energy needs, normal cells rely on respiration, which consumes oxygen and glucose to make energy-storing ATP. Cancer cells generally exhibit increased glycolysis for ATP generation (the Warburg effect) due in part to mitochondrial respiration injury and hypoxia [33]. Therefore, if cancer cells are reprogrammed and lose their tumorigenic potential, they will show a higher oxygen consumption and a reduction in glycolysis. ISL treatment decreased glucose uptake and lactic release. Furthermore, ISL reduced the mRNA expression of GLUT, HK2 and PFK-1. GLUT facilitates the transport of glucose, HK2 in turn mediates the first step of glycolysis, and PFK-1 regulates the rate-limiting step of glycolysis [34]. The inhibition of glycolysis was accompanied by depletion of ATP. Interestingly, we found an increased O_2_ consumption, a parameter of mitochondrial function, following ISL treatment. These observations suggest that ISL reverted metabolic and energy adaptations in A375 melanoma cells.

In many types of cancer, receptor tyrosine kinase (RTK) amplification and mutations, PIK3CA mutations and PTEN loss constitutively activate PI3K-AKT-mTOR signaling [35] and thereby reprogram cellular metabolism. mTOR is a serine/threonine protein kinase that integrates growth factor receptor signaling with cellular growth, proliferation and survival through two distinct multi-protein complexes. mTORC1, a validated cancer drug target, regulates protein translation through its substrates S6K1 and 4E-BP1 as well as anabolic metabolism downstream of growth factor receptor-activated PI3K-AKT signaling and in response to amino acid nutrient levels [36, 37]. However, mTORC2 is less well understood. Recent studies have suggested that mTORC2 may have an unexpectedly important role in cancer pathogenesis, promoting tumor growth and chemotherapy resistance in cancer cells, as well as controlling genome stability in yeast [38]. These effects appear to occur through AKT-independent signaling [39, 40]. mTORC2 is also necessary for the formation of EGFR-PI3K-driven gliomas in a Drosophila model [41], suggesting an important role for mTORC2 signaling that is independent of mTORC1-AKT activation. We sought to determine the impact of ISL on mTORC2 and measured the expression of the mTOR1-dependent protein RAPTOR in order to exclude the effect of mTOR1. We found that ISL modestly decreased the expression of RICTOR, whereas the expression of RAPTOR was not altered. A decrease in the phosphorylation of the downstream target AKT was also detected. AKT-catalyzed phosphorylation of another serine/threonine kinase, glycogen synthase kinase 3 (GSK3), results in GSK3 inhibition [42]. GSK3 is encoded by two known genes, GSK3 alpha (GSK3α) and GSK3 beta (GSK3β). GSK3β regulates a wide range of cellular processes including proliferation, energy metabolism and transcription control [42]. We found the expression of p-GSK3β to be suppressed after ISL treatment.

To determine the influence of mTORC2 on reprogramming of ISL-treated A375 cells, we used the mTOR-specific (mTOR1 and -2) inhibitor Ku-0063794. ISL co-treatment with Ku-0063794 induced a marked decrease in proliferation of A375 cells and an increase in melanin content and TYR activity. A significant reduction in glucose uptake and lactate release was also observed. A limitation of our study is that we were unable to find a mTOR2 specific inhibitor. However, our results identified mTORC2 as a central regulator of ISL-induced melanoma reprogramming and ruled out a role for mTOR1 in this process.

Here, we report that reprogramming of melanoma cells induced by ISL was responsible for diminishing cancer cell cachexia. The mTORC2-AKT-GSK3β signaling pathway has a central function in ISL-induced reprogramming. This work provides a new approach to induce solid tumor terminal differentiation and to investigate how metabolism alteration occurs in the re-differentiation of solid tumors. These findings open a new avenue for the treatment of melanoma.

## MATERIALS AND METHODS

### Chemicals

ISL (ISL, purity ≥ 98%) was purchased from Jiangxi Herb Tiangong Technology Co., Ltd. (Jiangxi, China). 3-[4,5-dimethylthiazol-2-yl]-2,5-diphenyltetrazolium bromide (MTT), 2,7-dichlorodihydro-fluorescein diacetate (DCFH-DA), dimethyl sulfoxide (DMSO), crystal violet were purchased from Sigma Chemicals (Sigma-Aldrich, St. Louis, MO, USA). Antibodies were purchased from Cell Signaling Technology (Danvers, MA).

### Cell culture

The human melanoma cell line A375 was obtained from Shanghai Biological Institute (Shanghai, China). Melanoma cells were cultured in Dulbecco's modified Eagle's medium (DMEM) supplemented with 10% fetal bovine serum, penicillin (100 mg/mL), and streptomycin (100 mg/mL) and incubated at 37°C in a humidified chamber using 5% CO_2_.

### Determination of cell proliferation parameters

Proliferation was determined by MTT assay [43]. Anchorage independent cell proliferation was evaluated using the colony formation assay [44].

### Apoptosis assay

Cells were treated with vehicle control or ISL and harvested by trypsinization after 24 h. Cells were incubated with Annexin-PI and the assay was carried out according to manufacturer's instructions (eBioscience, San Diego, CA). Cells were analyzed by flow cytometry using a BD FACS Calibur machine.

### Cell cycle analysis

Cell cycle analysis was performed using a kit (KeyGEN BioTECH, Nanjing, China) according to the manufacturer's instructions. Cells were processed by flow cytometry using a BD FACS Calibur machine.

### Determination of melanogenesis parameters

Melanin content in cell lysates was evaluated by spectrophotometry at 400 nm and expressed per mg of protein. Tyrosinase (TYR) activity was assayed by measuring L-3, 4-dihydroxyphenylalanine (L-DOPA) oxidase activity [26]. The dopachrome levels were measured at 492 nm [26].

### Glucose uptake

Glucose uptake experiments were performed using 2-NBDG (2-(N-(7-nitrobenz-2-oxa-1,3-diazol-4-yl)amino)-2-deoxyglucose) (Invitrogen, Carlsbad, CA, USA) according to the manufacturer's instructions. Briefly, cells were plated in a 96-well black clear bottom plate (Brand, Wertheim, Germany). After treatment, cells were washed three times with warm 1× PBS and incubated for 30 min in zero glucose DMEM containing 75 μM 2-NBDG, then washed three times with cold PBS. To each well, 200 μl of PBS was added and the relative fluorescence was measured using a fluorimeter (Synergy H1 multimode microplate reader; Biotek (Winooski, VT, USA); excitation 485 nm, emission 535 nm). The assay was normalized to the total amount of cellular protein.

### Lactate production

Lactate was measured in the cultured media using a Lactate Assay kit (Source Bioscience Life Sciences) according to the manufacturer's instructions. Cells were subsequently washed with cold PBS and lysed with 0.1 mol/L NaOH. Incorporated radioactivity was assayed by liquid scintillation counting and normalized to protein content.

### Determination of OCR

O_2_ consumption rate (OCR) was determined by high-resolution respirometry using an Oroboros Oxygraph-2 k instrument (OROBOROS^®^ INSTRUMENTS GmbH, Innsbruck, Austria). Cells (control or ISL-treated) were seeded at 4 × 10^6^ cells. The cells were centrifuged at 1000 rpm for 4 minutes and resuspended in MIR05 buffer (Oroboros lab). The respiration experiments were conducted at 37°C in MIR05 buffer. A standard protocol using malate (2 mM), glutamate (10 mM), oligomycin (2 μg/ml), FCCP (carbonyl cyanide p-trifluoromethoxyphenylhydrazone) (0.45 μM), succinate (10 mM), digitonin (3.68 μM), rotenone (0.5 μM) and antimycin A (2.5 μM) was used for each measurement. Cellular O_2_ was calculated from the recorded data as the time derivative of the oxygen content in the chamber; O_2_ concentrations were calculated using DatLab software (Oroboros Instruments).

### ATP assay

Total cellular ATP levels were determined by using an ATP assay kit (Roche, Indianapolis, IN, USA). ATP levels were determined following the manufacturer's instructions and normalized to total protein.

### qPCR

Total RNA (1 mg) was reverse-transcribed using TaqMan Reverse Transcription Reagent Kit. Measurement of gene expression was performed by quantitative real-time PCR (RT-PCR; ABI PRISM 7700 Sequence Detector, Applied Biosystems). The amount of target, normalized to an endogenous reference (eukaryotic 18S RNA, endogenous control, Applied Biosystems) was determined by using 2^−ΔΔCT^ calculation (Primers used for qPCR were showed in Supplementary Table 1).

### Western blotting

Whole-cell extracts were prepared using RIPA buffer (50 mM Tris-HCl, pH 8.0, 150 mM NaCl, 0.1% SDS, 0.5% sodium deoxycholate, 1% Triton X-100) supplemented with fresh protease and phosphatase inhibitors. 100 μg of each extract was resolved by gel electrophoresis on 8, 10, or 12% SDS-polyacrylamide. Western Lightning Plus ECL chemiluminescent reagent (Thermo Fisher Scientific, Waltham, MA) was used for detection of proteins. Imaging was performed using a GBOX system and protein band quantification was performed using Genetools software (Syngene, Frederick, MD). All proteins were normalized to a loading control.

### Statistical analysis

Data are presented as the mean ± SD from at least 3 independent experiments. Statistical analysis of the data was performed by Student *t* test. *P* values of 0.05 were considered statistically significant.

## SUPPLEMENTARY MATERIALS FIGURES AND TABLES



## Abbreviations

ISL: isoliquiritigenin
TYR: tyrosinase
L-DOPA: L-3, 4-dihydroxyphenylalanine
OCR: O_2_ consumption rate
ATP: adenosine triphosphate
OXPHOS: oxidative phosphorylation
mTORC2: mTOR Complex 2
MITF: microphthalmia-associated transcription factor
GLUT1: glucose transporter-1
HK2: glycolytic enzymes hexokinase II
PFK-1: phosphofructokinase
2-DG: 2-deoxy-D-glucose
