# Supplementary material for: Reprogramming induced by isoliquiritigenin diminishes melanoma cachexia through mTORC2-AKT-GSK3β signaling
Source: Oncotarget. 2017 Mar 29;8(21):34565–75. doi: 10.18632/oncotarget.16655 (PMC5470991; doi:10.18632/oncotarget.16655)
Supplement: Supplementary file 1 [file oncotarget-08-34565-s001.pdf]

Reprogramming induced by isoliquiritigenin diminishes melanoma cachexia through mTORC2-AKT-GSK3β signaling

Supplementary Materials

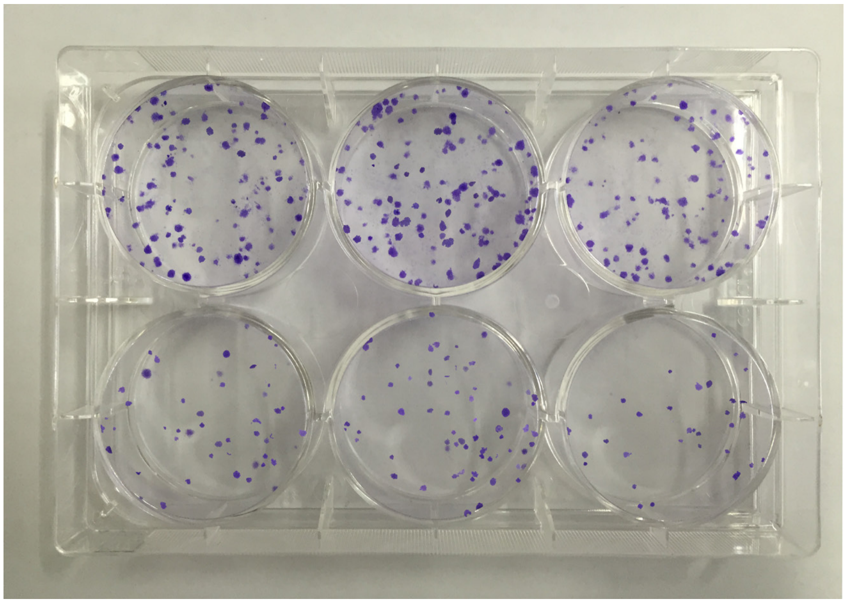

Ctrl

ISL
15μg/ml

Supplementary Figure 1: The colony formation assay was visualized by crystal violet-stained colonies.

Supplementary Table 1: Primers used for qPCR analysis

Gene	Primers
TYR	Forward primer: TACGGCGTAATCCTGGAAAC<br>Reverse primer: ATTGTGCATGCTGCTTTGAG
TYRP1	Forward primer: CCGAAACACAGTGGAAGGTT<br>Reverse primer: TCTGTGAAGGTGTGCAGGAG
MITF	Forward primer: CTCGAGCTCATGGACTTTCC<br>Reverse primer: CCAGTTCCGAGGTTGTTGTT
GLUT1	Forward primer: TCACTGTGCTCCTGGTTCTG<br>Reverse primer: CCTGTGCTCCTGAGAGATCC
HK2	Forward primer: TAGGGCTTGAGAGCACCTGT<br>Reverse primer: CCACACCCACTGTCACTTTG
PFK-1	Forward primer: CCCGTGTCTTCTTTGTCCAT<br>Reverse primer: GTTGTAGGCAGCTCGGAGTC
RICTOR	Forward primer: AATTGGAAAAGTGGCACAGG<br>Reverse primer: GGCAGCCTGTTTTATGGTGT
RAPTOR	Forward primer: CCCTGCTACTCGCTTTTGTC<br>Reverse primer: GTGAGGTGTTTCCCCTTTCA
GAPDH	Forward primer: CAAGGTCATCCATGACAACTT<br>Reverse primer: GTCCACCACCCTGTTGCTGTA
